# Performance of coumarin-derived dendrimer-based fluorescence-linked immunosorbent assay (FLISA) to detect malaria antigen

**DOI:** 10.1186/1475-2875-13-266

**Published:** 2014-07-10

**Authors:** Seon-Ju Yeo, Dinh Thi Huong, Jin-Hee Han, Jung-Yeon Kim, Won-Ja Lee, Ho-Joon Shin, Eun-Taek Han, Hyun Park

**Affiliations:** 1 Zoonosis Research Center, Department of Infection Biology, School of Medicine, Wonkwang University, Iksan, Jeonbuk 570-749, Republic of Korea; 2 Department of Medical Environmental Biology and Tropical Medicine, School of Medicine, Kangwon National University, Chuncheon, Gangwon-do, Republic of Korea; 3 Division of Malaria and Parasitic Disease, Korea National Institute of Health, Osong 363-951, Republic of Korea; 4 Department of Microbiology, Ajou University School of medicine, Suwon 443-721, Republic of Korea

**Keywords:** Coumarin-derived dendrimer, Fluorescence-linked immunosorbent assay (FLISA), Lactate dehydrogenase (LDH), ELISA, *Plasmodium falciparum*, *Plasmodium vivax*

## Abstract

**Background:**

Due to limitation of conventional malaria diagnostics, including microscopy, polymerase chain reaction (PCR), and enzyme-linked immunosorbent assay (ELISA), alternative accurate diagnostics have been demanded for improvement of sensitivity and specificity.

**Methods:**

Serially diluted *Plasmodium* LDH antigens, *Plasmodium falciparum*-infected human red blood cells (RBC) derived from *in vitro* culture or patient’s samples were used for evaluation of the performance of fluorescence-linked immunosorbent assay (FLISA). Microscopic examination was used to determine parasite density and the performance of FLISA was compared to ELISA. Finally, sensitivity and specificity of FLISA was determined by human specimens infected with *P. falciparum*, *Plasmodium vivax*, *Toxoplasma gondii*, and amoebae.

**Results:**

As a result of FLISA, the fluorescent intensity was highly correlated with antigen amount and FLISA was more sensitive than ELISA. FLISA detected at least 0.01 ng/ml of pLDH antigen, which showed 1,000-fold higher sensitivity than ELISA. *In vitro*-cultured *P. falciparum* was detected up to 20 parasite number/μL in FLISA but 5120 parasite number/μLin sandwich ELISA. *In vitro P. falciparum*-infected RBC number was highly correlated with fluorescent intensity (R^2^ = 0.979), showing that FLISA was reliable for detection of *P. falciparum* and available for quantification of parasite numbers. Furthermore, eighteen patient samples infected with *P. falciparum* (n = 9) and *P. vivax* (n = 9) showed 100% of sensitivity (18/18). FLISA showed 96.3% of specificity (26/27) because one sample of patient blood infected with *T. gondii* gave a false positive reactivity among healthy donors (n = 9), *T. gondii*-infected patients (n = 9), and amoeba-infected patients (n = 9).

**Conclusion:**

FLISA has a keen and high performance to detect malaria antigen, suggesting a potential assay as malaria immunodiagnostic.

## Background

Five human infectious malarias, including *Plasmodium falciparum*, *Plasmodium vivax*, *Plasmodium ovale*, *Plasmodium malariae,* and *Plasmodium knowlesi,* remain a serious health issue and are widespread in developing countries, continuing to cause about 219 million cases and one million deaths annually [1]. National malaria mortality rates are, however, particularly difficult to assess reliably and are underestimated in some important endemic areas due to misunderstanding malaria infection [2-4]. Therefore, the establishment of accurate diagnostics of malaria based on quantification of malaria parasite number with high sensitivity and specificity is the top priority for the management of malaria.

As a gold standard of malaria diagnostics, thick and thin blood smears are reliable but it was claimed that in the case of low parasitaemia, aggregation in a specific area of the smear was noticed and blood smearing was less sensitive than immunodiagnostics and PCR methods [5,6]. As an alternative quantitative diagnosis of malaria, over the last few decades, numerous polymerase chain reactions (PCR)-based diagnostic tests targeting RNA or DNA have been developed to confirm the malaria infection in addition to microscopic observation [7-9]. Quantification of malaria infectious parasite numbers in patients with only RNA or DNA copy numbers has been implemented but the requirement of equipped laboratory facilities presents an obstacle to application to a field setting [10]. Due to the limitation of PCR, different immunological assays that use antibodies to detect parasites have been developed with greater potential for adaption to field application than previous approaches, even though immune assays provide sensitivity issues [11]. Therefore, immunological assays have become the basis of most commercial diagnostic test kits, with most interest focused on the use of monoclonal antibodies (mAbs). Using mAbs, enzyme-linked immunosorbent assay (ELISA) method and fluorescence-linked immunosorbent assay (FLISA) have established significant diagnostic biomarkers [12,13]. Resulting devices for the diagnosis of malaria based on malaria-specific antigens, such as histidine-rich protein 2 (HRP2) and lactate dehydrogenase (LDH), have been developed as alternative diagnostics to microscopy and PCR [14].

Microscopy has often been the routine diagnostic technology available in developing countries. However, it has been considered to show variable sensitivity depending on expertise of microscopist [15]. Despite continuous application as key diagnostic tests, microscopy techniques have limitations as universal or targeted donor screening tests due to lack of sensitivity at low parasitaemia [16]. As a higher throughput method of malaria diagnosis, ELISA is suitable for epidemiological surveys [12].

Previously, performance of two monoclonal antibodies (D2H and D7E) targeting conserved 31 amino acids of *Plasmodium* LDH was shown to be potential to be useful for malaria diagnostics [17]. Besides, light-emitting diode (LED)-based novel organic fluorophore, coumarin-derived dendrimer was developed for malaria diagnostics [13]. As a light source of biosensor, LED has the advantage, because laser diode has various limitations such as expense, difficult operation, limited emission wavelength selections, and a short lifetime [13]. Therefore, development of LED light based diagnostics has advantage as further diagnostics. In this study, the enhanced coumarin-derived dendrimer-based malarial FLISA assay with novel monoclonal antibodies (D2H and D7E) was compared with ELISA.

## Methods

### *In vitro* culture and determination of parasitaemia

*Plasmodium falciparum* FCR-3 (ATCC 50005) were purchased from ATCC (Manassas, VA, USA) and strains used in this study were kept in -80°C as frozen stocks. *Plasmodium falciparum* was cultured by standard procedure as described previously [18], using a 5% haematocrit of type O-positive human red erythrocytes suspended in RPMI 1640 medium with 5% NaHCO_3_, 0.5% Albumax, 25 μg/mL gentamicin and supplemented with heat-inactivated 10% type O-positive human serum. The six-well plates were placed in an incubator (5% CO_2_, 5% O_2_, and 90% N_2_ atmosphere) at 37°C, and the medium was changed daily at least 5% parasitaemia. Blood smears were stained with Giemsa for counting parasite numbers with microscopy under 1,000 × magnifications. Parasite density was determined as a percentage of infected erythrocytes in fields of total 500 erythrocytes in the study. Animal experiments were performed at the experimental protocol, which was approved by the Animal Care and Use Committee at Wonkwang University.

### Purification of coumarin-derived dendrimer-or horseradish peroxidase (HRP)-bioconjugates

Subcloned cells (D7E or D2H) secreting pLDH were kindly provided by Professor Ho-Joon Shin in Ajou University, which were used in the previous report [17]. To obtain mouse ascitic fluid, 1 × 10^7^ cells were injected in incomplete Freund's adjuvant-primed ICR mice. Ascitic fluids harvested were further processed for purification of antibodies by protein A agarose 4B column (Incospharm, Korea) according to manufacturer’s instruction.

Purified D2H (1 mg/ml) were gently mixed with conjugation buffer (0.1 M NaHCO_3,_ pH 8.5) for 30 min at room temperature (RT) with gentle rotation. After coumarin-derived dendrimer (a kind donation of Dr Hak Sung Kim, Wonkwang University, Korea) was dissolved in DMSO (1 mg/ml), antibody (D7E) in conjugated buffer was incubated with 6 moles per mole of antibody for overnight at 4°C. Then the mixture was filtered by using desalting column (Thermo Scientific™ Zeba™ Spin Desalting columns) to remove free coumarin-derived dendrimer. Coumarin-derived dendrimer bioconjugate was collected with centrifugation at 1,000 × g for 2 min at 4°C.

D2H (1 mg/ml) was conjugated with horseradish peroxidase (HRP) in EZ-Link™ Plus Activated Peroxidase (Thermo Scientific, Rockford, IL, USA) according to manufacturer’s instructions. Briefly, 1 mg/ml of D2H in carbonate-bicarbonate buffer (pH 9.4) were reacted with 1 mg of lyophilized peroxidase at RT for one hour. After additional reaction with 10 μL of sodium cyanoborohydride for 15 min at RT, reaction stopped by 20 μL of quenching buffer for 15 min at RT. The HRP bioconjugates were further purified with conjugate purification kit (Thermo Scientific, Rockford, IL, USA) to remove unbound HRP.

### Fluorescence-linked immunosorbent assay (FLISA)

FLISA were performed as previously described [13]. *Plasmodium falciparum* and *Plasmodium vivax* recombinant LDH (rLDH) were purchased from C&K Bio Resource Inc, Korea. Briefly, 1 μg/well D7E were prepared for coating a black 96-well microtitre plate (Greiner, Germany) overnight and next day, plate was blocked with 2% casein-based blocking buffer at 37°C. After two hours, plate was washed with 200 μL of phosphate buffered saline with 0.05% Tween 20 (PBS-T, pH 7.4) and 100 μL of sample were added to each well and incubated at 37°C. After two hours, plate was washed with 200 μL of PBS-T. Finally, 200 μL of coumarin-derived dendrimer-conjugated D2H was added to detect antigens for one hour. Stringent washing with PBS-T was performed six times to remove unbound antibodies and 100 μL of PBS was added to each well to measure the fluorescence. Fluorescence with 460 nm excitation and 560 nm emission was measured using an Infinite F200 microplate reader (TECAN, Männedorf, Switzerland). Relative fluorescence was determined by extracting fluorescence value of negative control.

### Sandwich enzyme-linked immunosorbent assay (ELISA)

Polystyrene, 96-well plate (NUNC, Pasadena, Texas, USA) was coated with 100 μL (10 μg/mL) of D7E in coating buffer (50 mM bicarbonate/carbonate coating buffer, pH 9.6) and stored in an incubator overnight at 4°C. After incubation, the plate well was washed four times with 200 μL of PBS-T and blocked with 1% casein-based blocking buffer at 37°C for two hours. After washing with PBS-T buffer twice, differently diluted analytes were allowed to each well. The 100 uL of HRP conjugates D2H (2 ug/ml) was applied to each well at 27°C for one hour. After washing with PBS-T five times, plates were filled with 100 μL of 3,3’,5,5;-tetra methyl benzidine (TMB) substrate solution (Invitrogen, Camarillo, CA, USA) in the dark for 30 min, following stopping with additional 100 μL of sulphuric acid (0.5 M) per well. Optical density (OD) was measured by a microplate reader at 450 nm. Data were expressed as mean ± standard deviation.

### Ethical considerations

Professor Tai-Soon Yong (Yonsei University, College of Medicine, Seoul, Korea) kindly provided amoeba-infected specimens and Professor Ho-Woo Nam (Catholic University, College of Medicine, Seoul, Korea) kindly provided *Toxoplasma gondii*-infected samples. Malaria patient samples were provided from professor Eun-Taek Han (Kangwon National University, School of Medicine, Chuncheon, Korea). The study was approved by the Kangwon National University Hospital Institutional Review Board (Approval No. 10-041-07).

## Results

**Figure 1:**
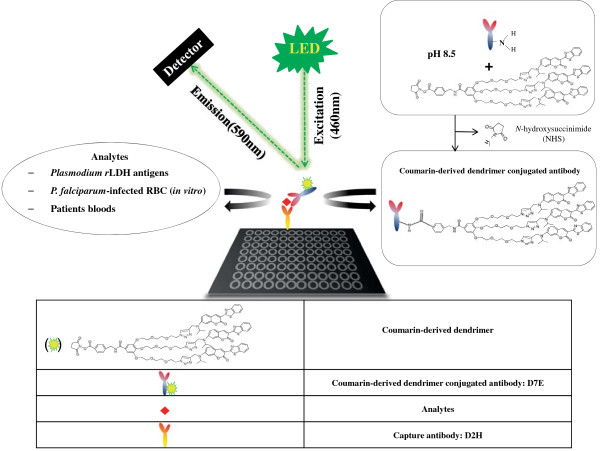
**Schematic illustration of FLISA with novel monoclonal antibodies targeting pLDH and coumarin-derived dendrimer.** LED-based FLISA consists of a capture antibody, analytes and coumarin-derived dendrimer conjugated mAbs. Plate was coated with capture antibody for overnight. Meanwhile, amine group of mAbs react with *N*-hydroxysuccinimide (NHS) of coumarin-derived dendrimer buffer (pH 8.5) to form stable amide bonds upon release of NHS. After washing, LDHs from analytes were detected by coumarin-derived dendrimer conjugated antibody and LED induced excitation of coumarin-derived dendrimer at 460 nm and emission at 590 nm were detected.

Coumarin-derived dendrimer was chosen as labelling fluorophore, which was confirmed to have good spectroscopic properties in previous study [13]. In this study, two newly developed mAbs targeting pLDH were used for FLISA instead of commercialized mAbs of pLDH, and performance in FLISA was defined to determine antigen-antibody reactivity. Figure 1A illustrates the schematic representations of the conjugation reactions to understand the principle of the coumarin-derived dendrimer-based FLISA with three analytes (*Plasmodium* recombinant LDH, *in vitro P. falciparum*-infected human RBC, and patients bloods). *N*-hydroxysuccinimide (NHS) esters of coumarin-derived dendrimers react with primary amine groups (-NH_2_) of mAbs in buffer (pH 8.5) to form stable amide bonds upon release of NHS. Further FLISA procedure is followed on conventional sandwich ELISA and in FLISA, fluorescence intensity indicates the interaction between parasite antigen and coumarin-derived dendrimer-conjugated antibody. The wavelength of maximum absorption (usually the same as the excitation maximum) of coumarin-derived dendrimer is 460 nm and excitation spectrum of a given bioconjugate is 590 nm [13].

*Plasmodium* LDH is an enzyme of glycolytic pathway of different *Plasmodium* species at both the asexual and sexual stages and also correlated with the number of parasites present in the plasma of infected patients [19-21]. The LDH from *P. vivax*, *P. malariae*, and *P. ovale* has 87% sequence identity with *p*LDH from *P. falciparum*[22]*.* In current study, target epitope (1–31 amino acid) of *Plasmodium* LDH were aligned in BLAST, using BLASTP 2.2.29+ program and the epitope sequence of *Plasmodium* LDH shows 97-100% identities among seven *Plasmodium* species [*P. falciparum* (GenBank accession no. AEX28368.1), *P. vivax* (GenBank accession no. AAS77573.1), *P. malariae* (GenBank accession no. AAS77572.1), *P. ovale* (GenBank accession no. AAS77571.1), *P. knowlesi* (GenBank accession no. AEL88505.1), *Plasmodium yoelii* (GenBank accession no. XP724101.1), and *Plasmodium berghei* (GenBank accession no. XP679401.1)]. In contrast, the eptitope of *Plasmodium* LDHs shows 76% identities of *Emeiria maxima* LDH (GenBank accession no. AAN38977.1) and 66% of *Toxoplasma gondii* LDH (GenBank accession no. XP002368488.1). This epitope has only 26% identities in *Homo sapiens* LDH (GenBank accession no. CAE11711) (Table 1). Therefore, antibodies targeting this epitope may act as pan-specific anti-LDH, which is able to react with most *Plasmodium* spp.

**Table 1:** **Alignment of pLDH epitope among *Plasmodium *** **species**

**Species**	**Epitope region in LDH**	**Identity**	**GenBank accession no.**
***P. vivax***	75 FTKAPGKSDKEWNRDDLLPLNNKIMIEIGGH 105	31/31(100%)	AAS77573.1
***P. falciparum***	61 FTKAPGKSDKEWNRDDLLPLNNKIMIEIGGH 91	31/31(100%)	AEX28368.1
***P. knowlesi***	82 FTKAPGKSDKEWNRDDLLPLNNKIMIEIGGH 112	31/31(100%)	AEL88505.1
***P. yoelii 17XNL***	82 FTKAPGKSDKEWNRDDLLPLNNKIMIEIGGH 112	31/31(100%)	XP724101.1
***P. berghei ANKA***	82 FTKAPGKSDKEWNRDDLLPLNNKIMIEIGGH 112	31/31(100%)	XP679401.1
***P. malariae***	75 FTKVPGKSDKEWNRDDLLPLNNKIMIEIGGH 105	30/31(97%)	AAS77572.1
***P. ovale***	75 FTKAPGKSDKEWNRDDLLPLNNKIMIEIGGH 105	31/31(100%)	AAS77571.1
***Eimeria maxima***	88 TKIPGKSDKEWSRMDLLPVNIKIMREVGG 116	22/29(76%)	AAN38977.1
***Toxoplasma gondii***	86 LTKVPGKSDKEWSRNDLLPFNAKIIREVA 114	19/29(66%)	XP002368488.1
***Homo sapiens***	98 AGARQQEGESRLNLVQRN123	7/31 (23%)	CAE11711

*Plasmodium* LDH epitope (FTKAPGKSDKEWNRDDLLPLNNKIMIEIGGH) is highly conserved among seven *Plasmodium* spp. and the percentage of sequence identity is determined by epitope alignments across the listed in NCBI.

**Figure 2:**
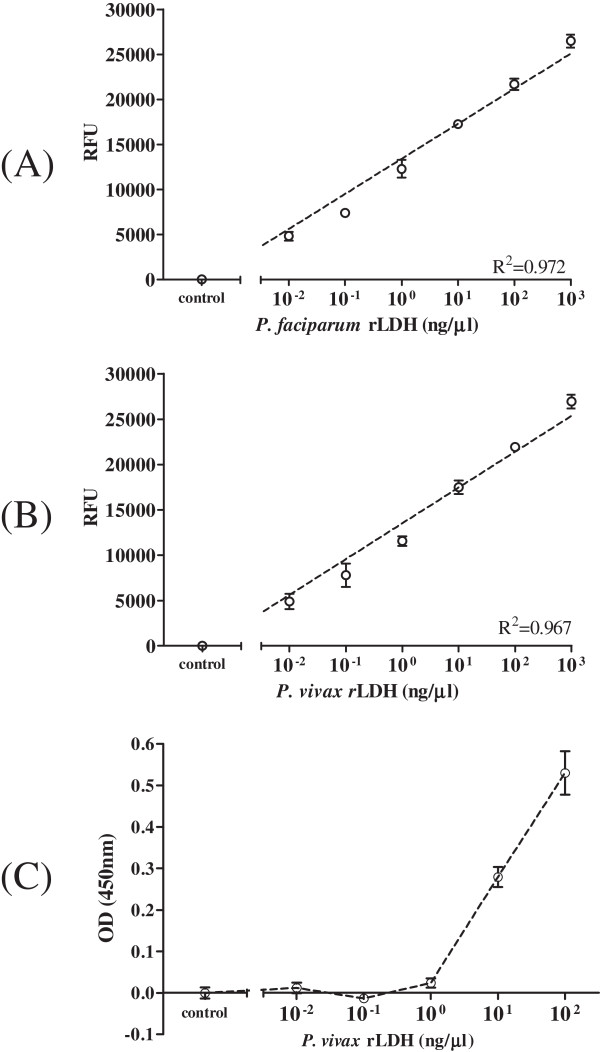
**Comparison of FLISA and ELISA detectability against *Plasmodium *LDH.** Standard curve for a detectable range of FLISA were generated: *P. falciparum* rLDH in FLISA **(A)**, *P. vivax* rLDH in FLISA **(B)**, and *P. vivax* rLDH in ELISA **(C)**. In FLISA, 0.01 ng/uL of antigen was detectable but 10 ng/uL of antigen was a minimum detectable antigen level in ELISA.

**Figure 3:**
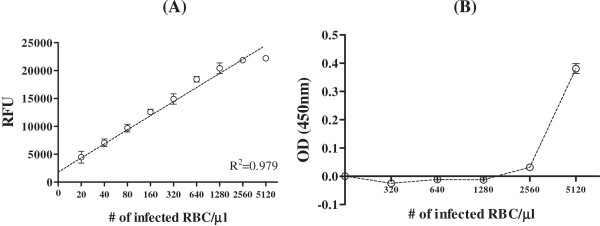
**Comparison of FLISA (A) and ELISA (B) analyses of *Plasmodium falciparum*-infected human red blood cells.** Human bloods were infected with *P. falciparum* FCR-3 for three days and when parasitaemia approached 3.2%, infected bloods were serially diluted and applied FLISA and ELISA.

To investigate the ability of FLISA to quantify malaria antigen, FLISA were performed with different concentration of *P. falciparum*- and *P. vivax*-recombinant LDH antigens at six-point standard curve based on two-fold dilutions from 0.01 to 1,000 ng/mL. Well-defined linear regressions were produced at FLISA for both *P. falciparum-*(R^2^ = 0.972) and *P. vivax* (R^2^ = 0.967) LDH (Figure 2A and B). *Plasmodium* LDH antigen was detectable up to 10 ng/mL in ELISA at the same condition with FLISA (Figure 2C). As OD value of 0.1 is positive, the detection threshold of FLISA is at least 1,000-fold higher than ELISA, implying that FLISA would contribute to increase detectability of pLDH in early infection when it is hard to be detected in conventional ELISA. Furthermore, *in vitro*-cultured *P. falciparum* was prepared to determine FLISA performance to detect *P. falciparum*-infected blood RBC. *P. falciparum* infected human RBC and cultured for three days to induce 3.2% of parasitaemia in 1.6 × 10^8^ total RBC. As a result of FLISA, fluorescent intensity and *P. falciparum* density was highly correlated and reliable (R^2^ = 0.97), between 20 and 5,120 parasites (Figure 3A). In contrast, detection threshold of ELISA was 5,120 parasites (Figure 3B). Therefore, FLISA would be a useful diagnostic assay with high detectability to quantify low number of parasite density than ELISA.

**Figure 4:**
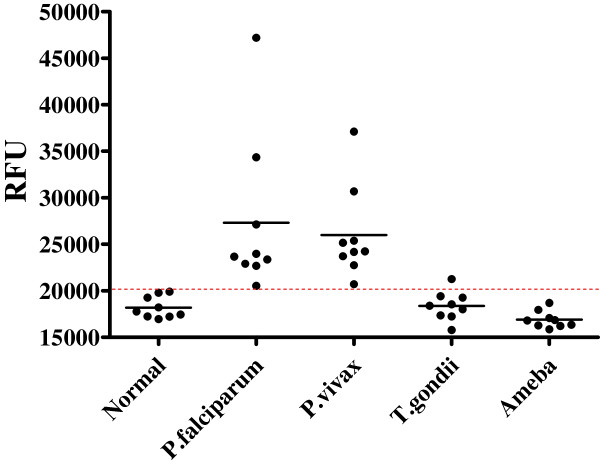
**FLISA performance to diagnosis malaria infection in patient samples.***P. falciparum* (n = 9)- and *P. vivax* (n = 9)-infected patients bloods were applied to FLISA to evaluate the sensitivity of FLISA. As non-malaria specimens, healthy donors (n = 9), *T. gondii* (n = 9)-, and amoeba (n = 9)-infected patient bloods were tested to determine the specificity. Red dotted line indicates the cut-off value between positive- and negative values of FLISA.

To determine the sensitivity and specificity of FLISA, *P. falciparum* (n = 9) and *P. vivax* (n = 9) infected patient’s bloods were applied to FLISA and FLISA showed 100% of sensitivity (18/18). In contrast, non-malaria disease samples among healthy donors (n = 9), *T. gondii*- (n = 9), and amoeba-infected patients (n = 9) showed one false positive reactivity with *T. gondii*-infected patient blood. Therefore, specificity of FLISA was 96.3% (26/27) (Figure 4).

## Conclusion

Since elimination of malaria infection by WHO, malaria prevalence is getting low but still prevalent worldwide. In Republic of Korea (ROK), after reemergence of malaria near location of demilitarized zone (DMZ) in 1993, the annual incidence has increased rapidly [23-25]. Until 2010, ten cases of transfusion-transmitted malaria have been reported in ROK [26]. The incidence of transfusion-transmitted malaria among people residing in endemic areas is unknown and a substantial proportion of the population in malaria-endemic countries has asymptomatic parasitaemia [16]. Therefore, development of enhanced *Plasmodium* blood screening test is advocated to prevent spread by transfusion in blood banks and in ROK, ELISA has been developed to screen and monitor transfusion-transmitted malaria [27,28]. There are two main aspects to keep in mind when considering malaria risk and transfusion, such as the malaria risk associated with any individual donor and the ability of the systems to identify and manage the donor [29]. The development of tests that are able to screen and identify donors potentially infectious for parasitic infections without causing the deferral of a large number of non-infectious donors is considered as one goal for prevention [30]. For this reason, coumarin-derived dendrimer-based FLISA assay was developed and compared to conventional ELISA. Because fluorescent density was highly correlated with parasite numbers, FLISA is applicable for quantification of parasite numbers in addition to diagnosis of *Plasmodium* infection. Therefore, FLISA has a potential as malarial diagnostic to facilitate the accurate diagnosis of *Plasmodium* infection and it would be an apparent alternative assay to determine *Plasmodium* parasite density. Because fluorescent intensity is correlated with malaria density with high sensitivity, FLISA may be considered as a feasible solution for high-throughput analysis of malaria infection during blood transfusion in ROK. Further studies should assess the diagnostic performance for other *Plasmodium* spp. infection and asymptomic parasitaemia patients.

## Competing interests

The authors have declared that they have no competing interests.

## Authors' contributions

SJ, DT, JH, JY, WJ, and HJ carried out the assays and analysed the results; SJ, ET and HP wrote the manuscript; SJ, WJ, HJ, ET, and HP conceived and designed the experiments. All authors read and approved the final manuscript.
